# Impact of genetics on third molar agenesis

**DOI:** 10.1038/s41598-018-26740-7

**Published:** 2018-05-29

**Authors:** Giedrė Trakinienė, Antanas Šidlauskas, Irena Andriuškevičiūtė, Loreta Šalomskienė, Vilma Švalkauskienė, Dalia Smailienė, Tomas Trakinis

**Affiliations:** 1 Lithuanian University of Health Sciences, Medical Academy, Department of Orthodontics, Kaunas, Lithuania; 2 Department of Genetics, Lithuanian University of Health Sciences, Institute of Biology Systems and Genetic Research, Kaunas, Lithuania; 3 Department of Orthopedic Surgery, Republican Hospital of Kaunas, Kaunas, Lithuania

## Abstract

The purpose of this investigation was to determine the impact of heritability on third molar agenesis in twins. The study sample consisted of 284 same sex twins (172 monozygotic and 112 dizygotic), whose mean ages were 19.7 ± 4.3 and 18.9 ± 4.8 years, respectively. The monozygotic group consisted of 36.3% males and 63.7% females, while the dizygotic group consisted of 50.1% males and 49.9% females. The zygosity of the twins was established using 15 specific DNA markers. The prevalence of third molar agenesis in monozygotic twins was 19.6%, which was higher than in the dizygotic twins group (15.50%) (p = 0.004). In both groups, third molar agenesis was more frequent in the maxilla than in the mandible (p = 0.000). Agenesis of the maxillary third molars was mostly affected by additive genetic factors (62–63%), with the common environment and the specific environment accounting for up to 25% and 13%, respectively. In contrast, agenesis of the lower third molars was associated with a higher additive genetic determination (81–83%), with the specific environment accounting for 17% to 19%. The study’s conclusion is that the formation of the third molars follicle is strongly controlled by additive genetic factors.

## Introduction

Third molars, or wisdom teeth, are the last teeth that erupt into the oral cavity. Interestingly, third molars are unique teeth, as their eruption time may vary from 17 to 24 years, depending on the ethnographic region and race^1^. Recently, discussions regarding the influence of the third molars on dentition have become highly important from several aspects, including their development, formation and retention and their effect on teeth crowding^2–4^. Consequently, understanding of the regulatory mechanisms of third molar development variability is of great clinical importance for making decisions on the timing of third molar surgical removal, autologous transplantation, orthodontic treatment planning and chronological age estimation for medico-legal purposes^5^.

In general, agenesis is considered to be the result of disturbances during the early stages of tooth development^6^. Furthermore, the prevalence of third molars agenesis was found to be considerably higher than for the agenesis of other teeth, varying from 10% to 41% among different countries^7,8^. The lowest values for the agenesis of these molars were found in black Africans and Indians, exhibiting a prevalence of 10–11% of the population^9,10^, while in the Iranian population, the prevalence rates for the third molar absence approached 34.8%^11^. The highest values of the third molar absence were reported in Koreans (41%)^12^. These large differences could be explained by different methodologies and different ethnical backgrounds^13^.

For analyzing the influence of genetics and environment on teeth agenesis, the most informative studies were performed using a classical twins study model, which could informatively present the effect of genetics. The earlier twin studies had some bias related to the difficulty in ensuring accurate zygosity determination. The situation changed when serum and enzyme polymorphism analyses were introduced^14^. However, even then, this method did not yield very high accuracy rates. In subsequent years, another method, which uses the highly polymorphic regions of DNA obtained from blood or buccal cells, has been used to determine the zygosity of twins with an accuracy rate greater than 95%^15^. However, a major limitation of twin studies is that they do not represent the whole society as a general unit^16^. Nevertheless, these studies are useful for the evaluation of the importance of genetics and environment on the manifestation of physical traits or certain illnesses^17^.

Previous studies showed that genetics played a crucial role in the agenesis of lateral central incisors or second bicuspids using dental casts^18,19^. However, these studies did not clearly reveal the influence of heredity on the formation of the third molars, which could be highly different from the other teeth because of their unique development^1^.

First, these teeth begin their development later. The formation of the third molar follicle begins approximately 7 years of age, exhibiting an unusually long developmental stage, and these teeth do not have a primary counterpart^20^. Second, evaluation of third molar agenesis needs a radiological evaluation of tooth germs, as they must attain a certain level of mineralization to be visible in the radiographs. Consequently, Rakhshan recommended that only individuals over 12 years of age could be used to analyze the formation of these teeth and for the precise determination of their mineralization stages^21^.

Finally, it would be interesting to determine whether genetics or environment is the dominant factor in the agenesis of the third molars. The hypothesis for this study was that the dominant factor for the agenesis of the third molars was genetics.

## Materials and Methods

The approval for the study was obtained from the Regional Biomedical Research Committee (No. BE-2-12). The study was conducted in accordance with relevant guidelines and regulations. Furthermore, informed consents were obtained from each participant and from the parents/guardians of any participant younger than 18 years old. Each participant was individually informed regarding the project upon the human subject information consent.

### Study group selection

Subjects were randomly selected from the National Twin Center and stratified according to zygosity. The study sample consisted of the same sex twin pairs with normal growth and development according to human growth curves using anthropometric measures, such as height and weight. Criteria for inclusion were the following: Caucasian twin pairs of the same sex, twins older than 12 years of age, no congenital anomalies or syndromes, no missing teeth, no teeth extractions, no previous orthodontic treatment and good quality pretreatment digital panoramic radiographs that were made at the first visit of the twin pair to the National Twin Center. The selected radiographs were not older than 5 years during the analysis. Exclusion criteria were twins younger than 12 years, the presence of craniofacial syndromes, a history of orthodontic treatment and poor quality panoramic radiographs. Digital panoramic X-rays were used to minimize the radiation dose following standard radiation safety principles. Every digital panoramic radiograph was evaluated by one orthodontist who had received additional training for teeth bud mineralization assessment in panoramic radiographs. The investigations were conducted twice in a 2-week interval for the evaluator’s calibration. Intra-examiner reliability was assessed by means of Kappa statistics, which showed perfect agreement (Kappa for all third molar agenesis was 1).

### Genetic assessment and determination

A genetic analysis was performed using maximum likelihood genetic structural equation modeling (GSEM). More recently, the use of highly polymorphic regions of deoxyribonucleic acid (DNA) derived from blood or buccal cells raised accuracy of zygosity in up to 90–95% of cases^17^. The more precise determination at the level of 99.99% requires analyze at least 15 highly polymorphic regions of DNA. Ming-Jie Yang found that with the DNA-based 15 STR analysis amplified in a multiplex PCR, the determination of the zygosity is not only cost and time saving but also shows greater sensitivity and precision than conventional methods^18^. Thus, the zygosity was determined using DNA tests with the polymerase chain reaction set for the amplification of short tandem repeats with 15 specific DNA markers (D8S1179, D21S11, D7S820, CSF1PO, D3S1358, TH01, D13S317, D16S539, D2S1338, D19S433, vWA, TROX, D18S51, D5S818 and FGA), as well as the Amel fragment of the amelogenin gene for comparison of the genetic profiles.

The twin studies, combined with structural equation modeling, can provide estimates of the proportion of the total phenotypic variation attributable to additive genetic effects, shared or common environments, and unique environmental components^15–17^. The data were analyzed with the the OpenMx package (http://openmx.psyc.virginia.edu) for the relative influences of the additive genetic factors (A), non-additive genetic factors (D), common or shared environments (C) and unique environment factors (E)^18–22^. Using Open Mx to apply sophisticated modelling methods to multivariate and longitudinal sets of dental data from twins offers great potential for clarifying how genetic and environmental factors influence development of the human dentition^16^. This analysis allows estimation of the significance of the different components of variance: the additive genetic factor (A), the shared environment (C), the non-additive genetic factor (D) and the unique environment (E). The Akaike information criterion (AIC) statistics and the difference in the chi-square (χ^2^) value relative to the change in degrees of freedom provided an indication of the models’ goodness of fit^23^. The most parsimonious model (lowest AIC value) to explain the observed variance was selected. Additive genetic factors (A) refers to the deviation from the mean phenotype due to the inheritance of a particular allele and this allele’s relative (to the mean phenotype of the population) effect on phenotype. Dominant genetic variance (D) involves deviation due to interactions between alternative alleles at a specific locus. The general environmental variance (C) is attributed to the nongenetic sources of variation between individuals that are experienced by multiple individuals in a population. This variance is typically the largest component of variance in populations in natural conditions. While specific environmental variance (E) is determined by the variation within replicated genetic lines, to obtain an estimate of general environmental variance, replicates of each of the genetic lines need to be assessed in each natural or experimental environment of interest^24^. However, this analysis should be estimated with caution, as it is prone to difference in methodologies (sampling differences, chronological age differences and zygosity determination, among others), which may limit the chances of finding diverging models^24–27^.

### Statistical analysis

Statistical analyses were performed using SPSS Statistics for Windows, Version 17.0. The hypotheses of interrelations between characteristics were verified using the chi-square criterion and Spearman correlation coefficients (r). The justification that correlations between monozygotic twins are of a different degree than those in dizygotic twins was assessed by checking for the significance of difference between correlations using ANCOVA. A p-value smaller than 0.05 was considered to be significant. The post hoc analysis of the power of the study showed a level of 0.8.

### Data availability statement

The data will not be available for protection of the twins rights.

## Results

The study sample consisted of 284 twins (172 monozygotic and 112 dizygotic) whose mean ages were 19.7 ± 4.3 and 18.9 ± 4.8 years, respectively. The monozygotic group consisted of 36.3% males and 63.7% females, while the dizygotic group consisted of 50.1% males and 49.9% females.

The prevalence of third molar agenesis in monozygotic twins was 19.6%, which was higher than in the dizygotic twins group (15.50%) (p = 0.004). Females had a tendency to have third molar agenesis more frequently than the males in both groups, but these differences were likewise statistically insignificant (Table 1).

**Table 1:** Distribution of third molar agenesis according to sex, jaw and right-left side. (MZ - monozygotic twins, DZ - dizygotic twins, statistically significant results according to Fisher’s exact test: (p = 0.001)**.

Variables		Agenesis of third molar					
		DZ		MZ		All twins	
		Mean (%)	p	Mean (%)	p	Mean (%)	p
Sex	Males	5.90	0.651	7.30	0.569	7.70	0.689
	Females	9.60		12.30		9.80	
Jaw	Maxilla	9.30	0.000**	12.30	0.000**	10.8	0.000**
	Mandible	6.20		7.30		6.70	
Laterality	Right	7.30	0.091	11.2	0.067	9.3	0.061
	Left	8.20		8.4		8.2	

In both groups, third molar agenesis was more frequent in the maxilla than in the mandible, and these findings were statistically significant. Moreover, in monozygotic twins, this agenesis in the maxilla was approximately 59% higher than in the mandible, while in the dizygotic twins, upper third molar agenesis exceeded lower third molar agenesis by only 33%.

The analysis of the third molar laterality revealed that the prevalence of third molar agenesis had a tendency to be more common on the right side, especially in the monozygotic twins, but these differences were not statistically significant.

The distribution of third molar agenesis between siblings in monozygotic twins was nearly the same, while in dizygotic twins, these differences between the pair members were statistically significant (Table 2). The positive correlations of third molar agenesis among siblings were observed to be strongest in the monozygotic group, reaching 0.80, while in the dizygotic group, correlations reached only up to 0.25 which is rather weak and leaves a lot of space for other possible influences, causes and relations. These differences were found to be statistically significant (Table 3).

**Table 2:** Distribution of third molar agenesis between twin members in the twin pair. (MZ = monozygotic twins, DZ = dizygotic twins, statistically significant results according to Fisher’s exact test: (p < 0.05)*.

Variables	Intra-pair twin members	Distribution of third molar agenesis			
		DZ		MZ	
		Mean frequency (%)	p	Mean frequency (%)	p
18 tooth	Twin A	2.00	0.039*	3.80	0.457
	Twin B	3.10		3.40	
28 tooth	Twin A	1.50	0.039*	2.30	0.557
	Twin B	2.70		2.80	
38 tooth	Twin A	1.70	0.041*	1.90	0.668
	Twin B	2.30		1.40	
48 tooth	Twin A	1.40	0.048*	2.00	0.735
	Twin B	0.80		2.00

**Table 3:** Spearman correlations of third molar agenesis between siblings in dizygotic and monozygotic twins; ANCOVA test(significance of differences dizygotic vs monozygotic twins) statistically significant results: **p < 0.001.

Variable	tooth 18	tooth 28	tooth 38	tooth 48
Spearman correlations between siblings in dizygotic twins	0.25	0.24	0.21	0.22
Spearman correlations between siblings in monozygotic twins	0.57	0.55	0.79	0.80
ANCOVA test	p = 0.000**	p = 0.000**	p = 0.000**	p = 0.000**

The AIC values for each model were calculated and the most parsimonious models with the lowest values were chosen. Only the results of the best-fitting model have been taken into account. Variables with best fitting-model and contribution of factors (*a*^2^, *c*^2^, *d*^2^, *e*^2^) were counted^18^. The main paths of interest are those which get a start value of 0.5^20^. The analysis of these findings with the Open Mx program showed that the best fitting model for the agenesis of the maxillary third molars was ACE(AIC value was −8.5); however, in the mandible, a higher significance was seen using the AE model(AIC values was −9.05). The agenesis of the maxillary third molars showed the highly additive genetic determination *a*^2^, which accounted for 62 to 63% of the total variation, with the common environment accounting for up to 25% and the specific environment accounting for 13%. In contrast, the agenesis of the lower third molars was associated with higher additive genetic determination (81–83%), while the specific environment accounted for the remaining 17 to 19% (Table 4).

**Table 4:** Path coefficients for the agenesis of third molars (a^2^- additive genetic factors, d^2^- non-additive genetic factors, c^2^-common or shared environment, e^2^-specific environment factors); SE- standard error. Values in bold: path coefficients greater than 0.50 are significant.

Variable	Model	Genetics				Environment			
		a^2^	SE	d^2^	SE	c^2^	SE	e^2^	SE
Agenesis of 18 tooth	ACE	0.63	0.06			0.24	0.03	0.13	0.04
Agenesis of 28 tooth	ACE	0.62	0.07			0.25	0.05	0.13	0.04
Agenesis of 38 tooth	AE	0.81	0.04					0.19	0.06
Agenesis of 48 tooth	AE	0.83	0.05					0.17	0.02

## Discussion

This study tested the hypothesis that genetics plays a key role in the agenesis of the third molars using the genetic model of twins. Martin *et al*. have pointed out that usually before starting to look for quantitative trait loci (QTLs) for complex traits, it is worthwhile to show that there is a significant component of genetic variation present^28^. The results of our study revealed several findings that were different from other studies.

First, examination of the effect of sex on the agenesis of the third molar has been the subject of debate in previous studies. Kruger *et al*. revealed that females had higher values of third molar agenesis than males^29^. In contrast, the present study showed that the frequency of the third molar agenesis in males and females was almost the same, and these results were in agreement with a study from Alam *et al*., which reported that third molar agenesis was not influenced by sex^30^. This could be due to the differences in the populations.

Moreover, the previous findings in the evaluation of the distribution of the missing teeth in the maxilla and in the mandible are confusing. Sisman reported that maxillary lateral incisors were the most frequently absent teeth (27.95%)^31^. In contrast, the study by Chung reported that the agenesis of the mandibular second bicuspid was the most frequently observed in Koreans^32^. Also, a recent study showed that heritability estimates for the third molars had a tendency to be higher in the maxilla than in the mandible.

In addition to these findings, Sujon *et al*. revealed that third molar agenesis was more common on the right side than on the left^6^. This tendency was also observed in our study but did not have statistical significance and was in agreement with the study of Silva Meza R., who did not find any differences between missing teeth on the right and left sides^33^. These disparities may reflect different environmental effects.

Furthermore, while analyzing the localization of the missing teeth, Fekonja suggested that genes could be the dominant factor for the agenesis in the anterior region, while the posterior teeth could be missing sporadically^7^. Interestingly, the structural equation model, which was used in the current study for the calculation of the proportion of the total phenotypic variation attributable to additive genetic effects (narrow-sense heritability), showed that additive genetic determination of the third molar agenesis was the dominant factor. The significance of the heredity in the maxilla was lower than in the mandible. Perhaps the development of these teeth could be affected by several genes and possibly by their interaction with environmental factors as it is seen with other teeth^16^. Kangas *et al*. found that dental characters seem to be non-independent and that increasing the levels of expression of just one gene can lead to increases in cusp number, altered cusp shape and position, development of longitudinal crests on teeth, and increases in tooth number in experimental mice^34^. At the moment, these genes are not clearly identified, and this could be a good field for future investigations.

Previous studies support the view that, even though there is a relatively strong genetic basis to other missing teeth, the number or position of affected teeth can be influenced by epigenetic factors^35^. However, the precise nature of these influences is still unclear and they may be due to factors other than differences in methylation of DNA or acetylation of histones. Townsend *et al*. proposed that they could reflect different responses of odontogenic cells to minor variations in the spatial and temporal expression of local signalling molecules passing between cells during development and can lead to quite major differences in the final appearance of the dentitions^36^.

From a clinical perspective, knowledge about the development of the teeth could be highly useful for the understanding of the processes in the oral environment and for the preparation of the potential preventive features that could help patients with such pathology avoid different treatment difficulties.

In conclusion, the hypothesis that genetics plays the dominant role in the formation of third molars was supported.

### Limitations of The Study

Dental development is a multilevel process involving interacting genetic, epigenetic and environmental factors over an extended period. The precise analysis of the relationships of these factors warrants longitudinal studies on larger groups. In addition, the underlying assumption of twin studies is that their results can be extrapolated to the singleton population, but this possibility has not been definitively supported.

## Conclusions

This recent study showed that agenesis of the third molar was strongly affected by additive genetics. However, future studies on larger groups should be conducted to assess the role of genes, epigenetics and environment more precisely.
